# Modelling of forefoot injuries caused by brake pedal loading – a finite element analysis case study

**DOI:** 10.1186/1757-1146-7-S1-A59

**Published:** 2014-04-08

**Authors:** Bisola Mutingwende, Robert Ashford, Clive Neal-Sturgess, Maxine Lintern, Jens Lahr

**Affiliations:** 1 Centre for Health and Social Care Research, Birmingham City University, UK; 2 Department of Mechanical Engineering, The University of Birmingham, UK; 3 Faculty of Technology, Engineering and the Environment, Birmingham City University, UK

## Introduction

Lower extremity injuries, in particular the foot/ankle are one of the most common in automotive crashes.[1] Although not life threatening, they can lead to long term medical complications or permanent disability[²]. In most cases foot and/or ankle fractures are caused during frontal automotive crashes, while the driver attempts an emergency brake and the foot is subject to crash loading [³]. In these cases, fractures of the forefoot, in particular the metatarsals are very common [4] and range from simple fractures to severe crush injuries [5]. The location of metatarsal fractures in a car crash victim can be dictated by the loading pattern [5]. However, there is little information about whether the pedal has an effect on the mechanism or extent of injury. In order to evaluate the effect of brake pedal loading on the injury tolerance of the metatarsal, a computer based finite element analysis was performed to assess the regional capabilities in terms of loading transmission around the forefoot.

## Methods

**Figure 1:**
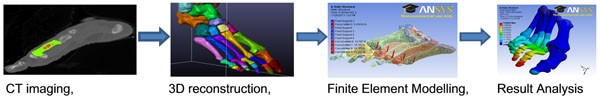
Flow chart of method

A Finite Element (FE) model of the foot and ankle was developed from a 3D reconstruction of CT images [6] of a female subject using Simpleware (Simpleware^®^ Ltd) segmentation software. The model was then imported into ANSYS Workbench for FE analysis (ANSYS^®^ Academic Research, Release 14.0). Material properties for the analysis were assumed to be homogenous and linearly isotropic. The cortical and simple ligamentous structures attaching the bones together were modelled. Variable pedal forces obtained from automobile crash data were applied to the forefoot region (i.e. ball of the foot) of the model, and the loading patterns of stress were analysed (Figure 1).

## Results

**Figure 2:**
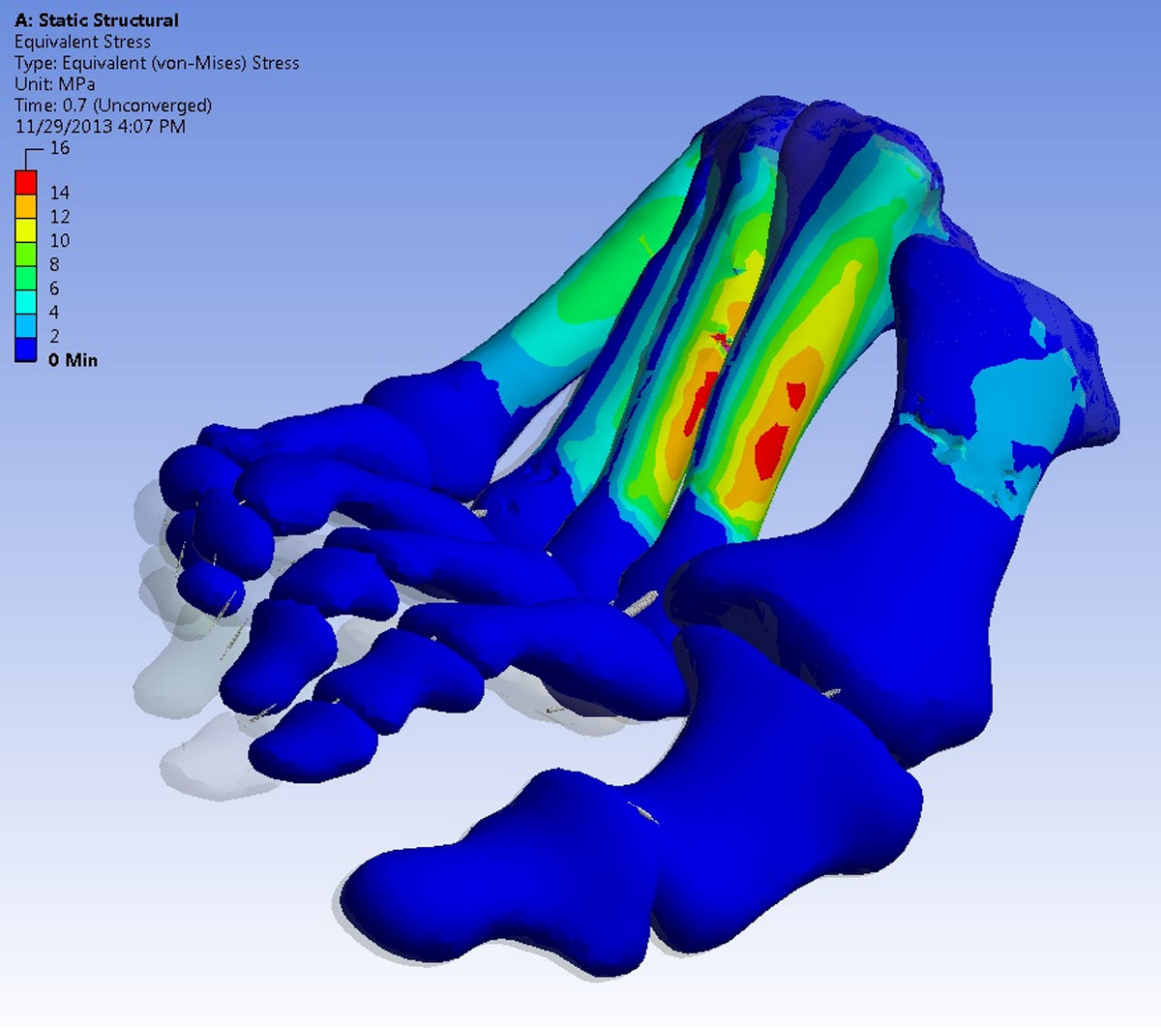
Equivalent Stress Distribution

The maximum stresses and angles of deflection were obtained from applying a range of pedal forces (2kN-10kN). The resultant predicted loading patterns for the metatarsals were then analysed (Figure 2). The highest stresses were found at the smallest cross-sections of the metatarsals. Although bending stresses increase with increasing distance from the point of load application, the larger cross-sections compensate for this effect.

## Conclusion

The results of the current study show that the locations of the maximum stresses appear on the second and third metatarsals. This compares with findings from crash data.

## References

[B1] Crandall JR Martin PG Sieveka EM Pilkey WD Dischinger PC Burgess AR O’Quinn TD Schmidhauser CB Lower Limb Response and Injury in Frontal Crashes Accident Analysis and Prevention 1998 30 5 667 677 10.1016/S0001-4575(98)00006-2 9678220

[B2] Dischinger PC Read KM Kufera JA Kerns TJ Burch CA Jawed N Ho SM Burgess AR Consequences and Costs of Lower Extremity Injuries AnnuProcAssocAdvAutomot Med 2004 48 339 53 PMC3217424 15319134

[B3] Neale M Thomas R Bateman H Hynd D A Finite Element Modelling Investigation of Lower Leg Injuries TRL Limited 2012 07 0077

[B4] Mihai-Costin C Injuries of the Foot and Ankle Joint and Their Mechanisms Technical paper for students and young engineers, University for Medicine and Pharmacy “IuliuHatieganu” Cluj-Napoca- Fisita - World Automotive Congress, Barcelona 2004

[B5] Arangio GA Beam HN Kowalczyk G Salatht EP Analysis of stress in the metatarsals Foot and Ankle Surgery 1998 4 123 128 10.1046/j.1460-9584.1998.00104.x

[B6] Visible Human Project® (National Library of Medicine, National Institutes of Health, 8600 Rockville Pike Bethesda, MD 20894)

